# Meta-Analysis of Saturated Fatty Acid Intake and Breast Cancer Risk

**DOI:** 10.1097/MD.0000000000002391

**Published:** 2015-12-31

**Authors:** Hui Xia, Shushu Ma, Shaokang Wang, Guiju Sun

**Affiliations:** From the Department of Nutrition and Food Hygiene, School of Public Health, Southeast University, Nanjing, P.R. China.

## Abstract

The associations between saturated fatty acid (SFA) consumption and risk of breast cancer (BC) remains inconclusive. Therefore, we conducted this meta-analysis to determine the quantitative relations between dietary SFA intake and incidence of BC.

Literatures published up to April 2015 were systematically screened through Pubmed and Web of Science. Relevant publication quality was evaluated by conducting the Newcastle-Ottawa scale. We used fixed effects models or random effect models to calculate the summary relative risks (RRs) and odds ratios (ORs), and conducted sensitivity analyses and evaluated the publication bias.

We identified a total of 52 studies (24 cohort studies and 28 case–control studies), with over 50,000 females diagnosed with BC. The associations between dietary SFA intake and risk of BC were 1.18 for case–control studies (high vs low intake, 95% confidence interval [CI] = 1.03–1.34) and 1.04 for cohort studies (95% CI = 0.97–1.11). When restricted analyses to population-based studies, positive associations were observed for both cohort (RR [95% CI] = 1.11 [1.01–1.21]) and case–control studies (OR [95% CI] = 1.26 [1.03–1.53]). Additionally, for case–control studies, significant positive associations between higher SFA intake and BC risk were observed for Asian (OR [95% CI] = 1.17 [1.02–1.34]) and Caucasian (OR [95% CI] = 1.19 [1.00–1.41]), as well as for postmenopausal women (OR = 1.33, 95% CI: 1.02–1.73). In contrast, higher dietary SFA intake was not associated with risk of BC among premenopausal women, in cohort studies or hospital-based studies.

A positive association between higher dietary SFA intake and postmenopausal BC risk was observed in case–control but not in cohort studies. More studies are warranted to confirm these findings.

## INTRODUCTION

The incidence of breast cancer (BC) ranked second among women worldwide,^1^ as well as in China,^2^ with a 3% increase per year.^3^ To date, World Health Organization/Food and Agriculture Organization recommends that the total saturated fatty acid (SFA) intake should be controlled within 10% of total daily energy for adults.^4^ Among the dietary SFAs, the common groups are myristic acid, palmitic acid, and stearic acid, which may be involved in the regulation of raising low-density lipoprotein-cholesterol and high-density lipoprotein-cholesterol.^4–6^

However, the relationship between dietary SFA intake and the incidence of BC remains uncertain. For example, the meta-analysis conducted by Boyd et al^7^ indicated a positive association between higher intake of SFA and BC risk (n = 34; highest vs bottom category; relative risk [RR] = 1.19; 95% confidence interval [CI]: 1.06–1.35). In contrast, Smith-Warner et al^8^ reported null associations (n = 8; highest vs bottom quartile; RR = 1.01; 95% CI: 0.89–1.16). Moreover, a cohort study in Japan was performed. And the total mortality of Japanese women was inverse to the intake of SFA (hazard ratio [HR] [95% CI] = 0.91 [0.83–1.00]).^9^ Due to different races background, people have varied eating habits. And menopausal processes and postmenopausal endocrine events can affect the development of BC.^10^ Nevertheless, none of the aforementioned publications included any subgroup analyses, such as ethnicity, population (hospital)-based females, menopausal status, and so on. Hence, the objective of the present study was to further investigate the association between dietary SFA intake and the incidence of BC with the more detailed analyses among observational studies.

## MATERIALS AND METHODS

We followed the Meta-analysis of Observational Studies in Epidemiology (MOOSE) guidelines^11^ to conduct this meta-analysis. Ethical approval was not necessary, because all publications included in this study were published officially.

### Literature Search

We systematically searched the literatures published in English in Pubmed, Web of Science up to April 2015, using search terms: (“dietary fat” or “saturated fatty acid,” or “saturated fat”) and (“breast” or “mammary”) and (“tumor” or “carcinoma” or “neoplasm”). The search was restricted to human studies. Reference lists from each study, systematic reviews and meta-analyses were reviewed to identify potential relevant literatures as well.

### Inclusion and Exclusion Criteria

Two investigators independently reviewed these studies. Studies were included when the following criteria were met: published openly; evaluated the association between SFA intake from food and the incidence of female BC only; specified diagnosis of BC; contained odds ratios (ORs), RRs, or HRs with corresponding 95%CIs or data could be estimated; and selected when data were most sufficient if they were from the same population. Studies were excluded when they were: animal or vitro experiments, review articles, repeated literatures, or mechanism studies; not related to human subjects; not of appropriate control groups; without analysis method provided; and were excluded when lack of access to full texts.

### Data Extraction and Quality Assessment

We obtained from each study the information on author's name, publication year, country, community, or study design based on hospital, dietary assessment method, and outcomes (RRs/ORs/HRs [95% CIs]). Ethnicity was classified as Asian and Caucasian. ORs, RRs, or HRs were extracted only when articles employed adjusted models with most confounders in original publications. The estimates from 1 study were recorded as much as possible including premenopausal and postmenopausal population. Newcastle-Ottawa Scale^12^ was used to evaluate quality of literatures independently by 2 investigators. The literatures with scores ≥5 were included in the meta-analysis.

### Statistical Analyses

We used STATA (version 11.0, StataCorp, College Station, TX) to perform the meta-analysis. We used RR as an approximate for HR in cohort studies. First, adjusted ORs or RRs comparing highest versus lowest category of dietary SFA intake were gathered with the corresponding 95% CIs as possible and meanwhile were calculated by the logarithmic transformation of RRs and ORs with the corresponding 95% CIs. As described in previous study,^13^ the fixed-effects model was used when I^2^ was lower than 50% and *P* of the value of heterogeneity was ≥0.05. Otherwise we used the random-effects model. Second, we conducted subgroup analyses by ethnicity (Asian, Caucasian), menopause status (premenopause, postmenopause), and study type (population, hospital-based). Finally, Begg funnel-plot and Egger test were conducted to examine publication bias with significance when the value of *P* is <0.05.^14,15^

## RESULTS

### Literature Search and Study Characteristics

**FIGURE 1:**
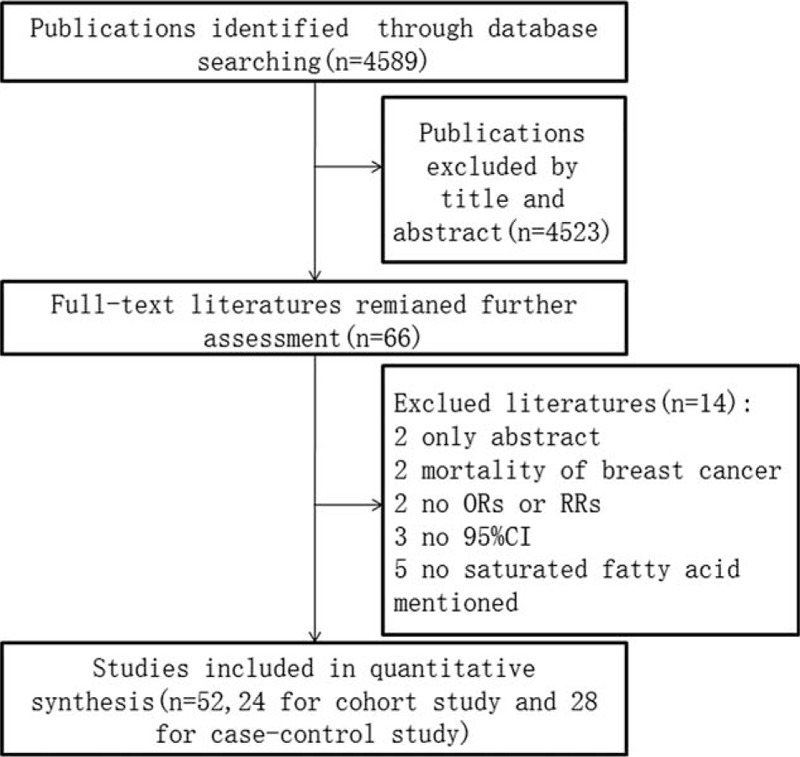
Flow diagram for selected articles (case–control and cohort study).

The flow chart for selected articles was shown in Figure 1. A total of 4589 publications were found through electronic search after removing those duplicates. A total of 4523 articles are most reviews, animal and vitro experiments. Finally, 52 articles (24 cohort studies^16–39^ and 28 case–control studies^40–67^) were eligible for this meta-analysis after checking the full text while 14 articles^68–81^ were excluded for additional reasons.

Characteristics of 52 studies were shown in Table 1 . Among cohort studies, a total number of 1,786,537 subjects had been followed up ranging from 3.3 to 20 years with 35,651 diagnosed with BC. Among case–control studies it contained 17,015 cases and 22,192 controls. Food frequency questionnaires were most frequently used to evaluate dietary SFA intake. Information of 35 studies included was from community only, information of 16 studies was from hospital simply and 1 was from both community and hospital. Eight studies were reported from Asian only, 40 were from Caucasian simply, and 2 were from Caucasian and Asian. Newcastle-Ottawa Scale scores of all studies ranged from 5 to 8 and 96.2% publications’ scores were ≥6.

**TABLE 1:**
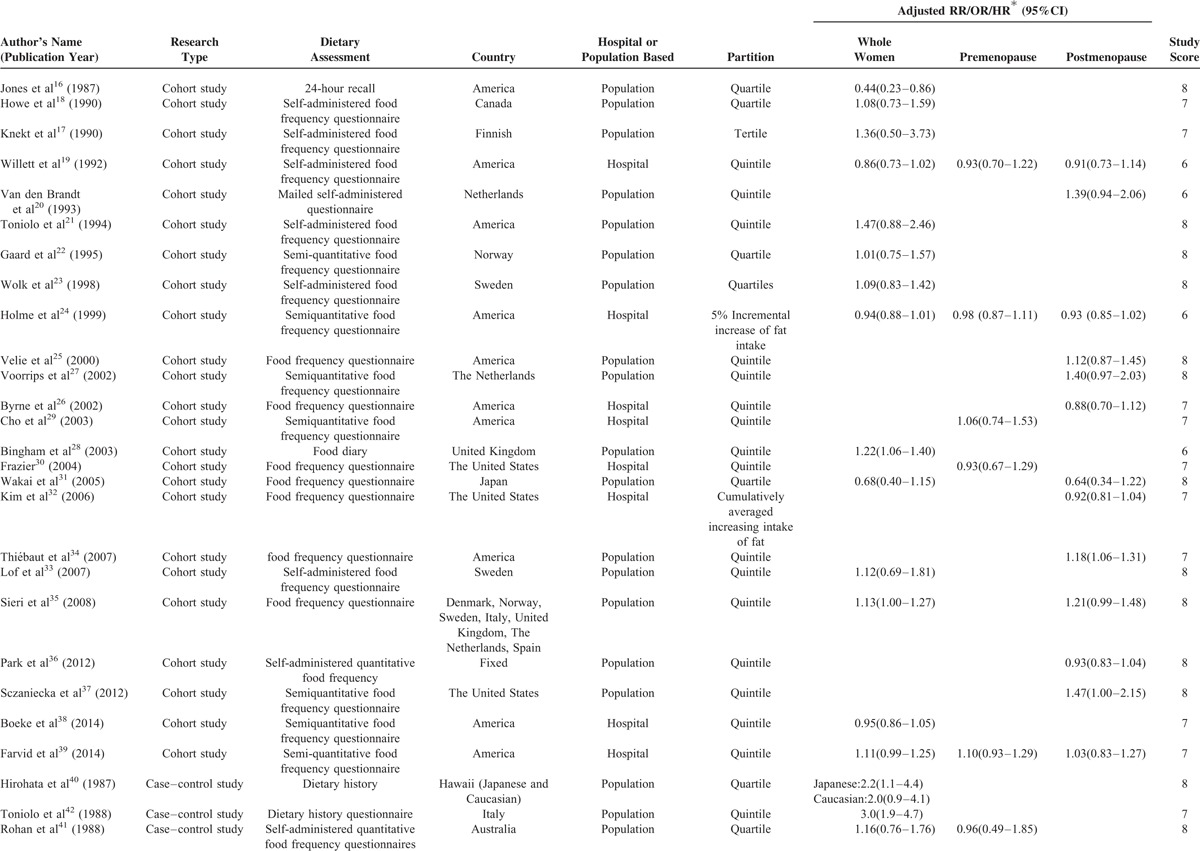
Basic Information of Included Studies

**TABLE 1 (Continued):**
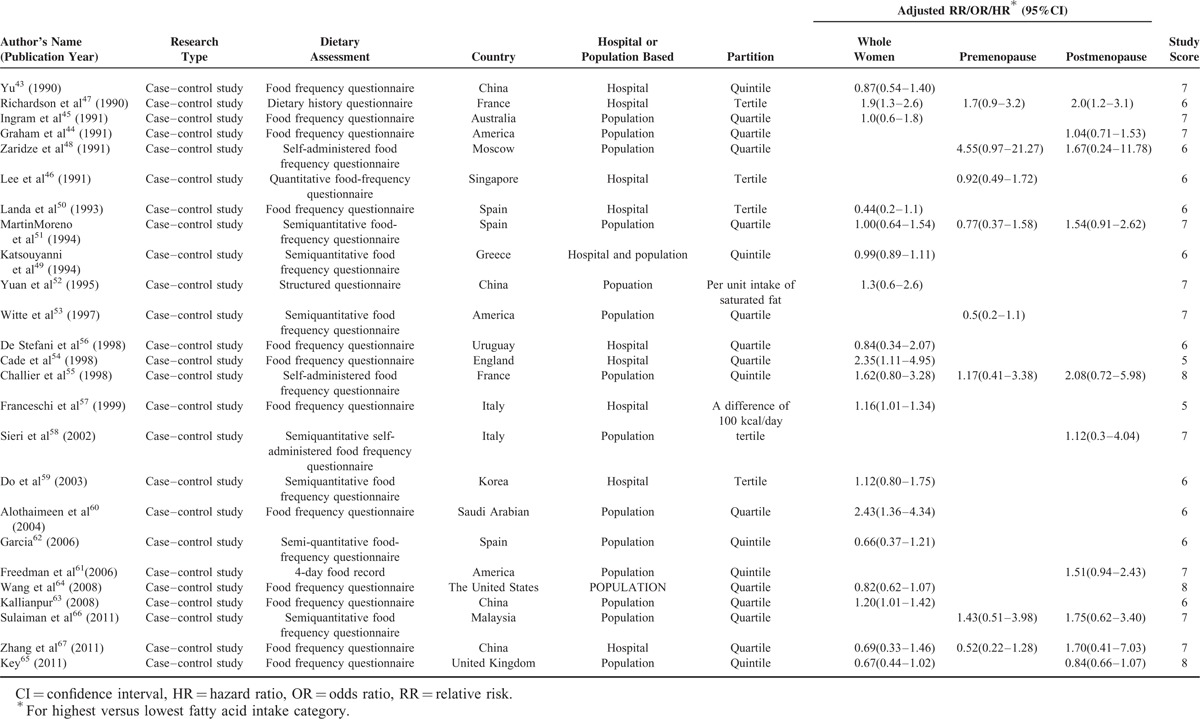
Basic Information of Included Studies

### Highest Versus Lowest Intake of Meta-Analysis

We analyzed cohort and case–control studies separately owing to the relatively higher incidence of BC.

**FIGURE 2:**
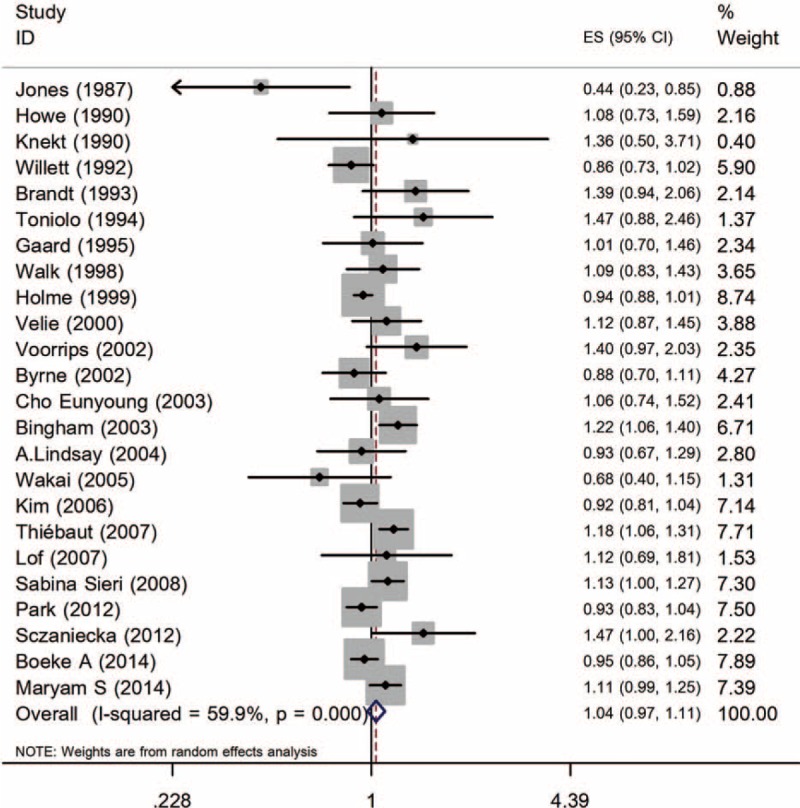
Forest plot for cohort studies.

**FIGURE 3:**
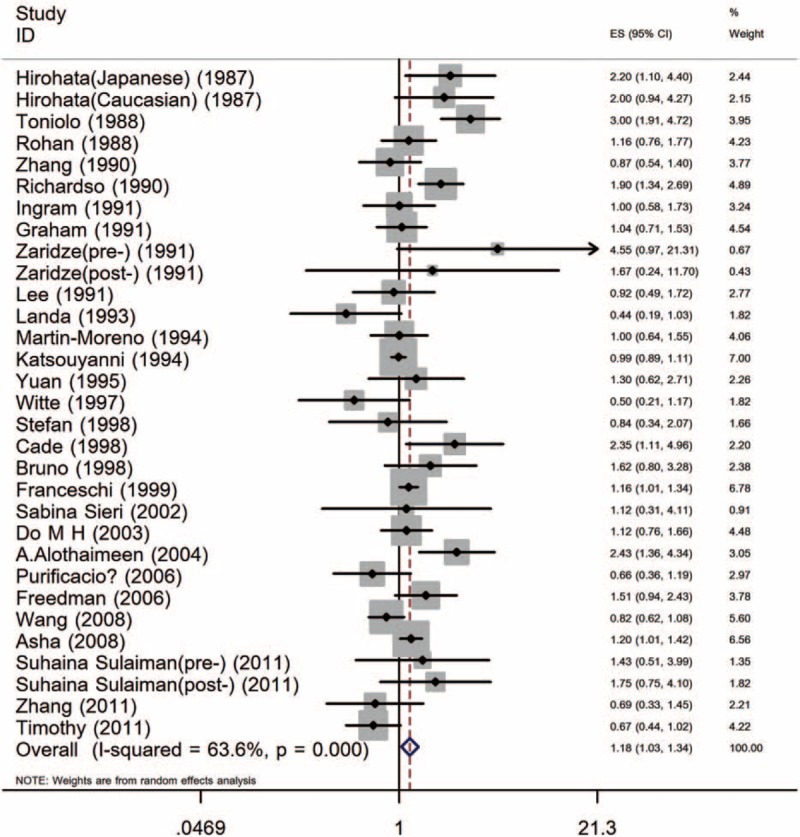
Forest plot for all case–control studies.

The forest plots of 52 studies together were shown in Figures 2 and 3. Intake ratio of dietary SFA was not associated with BC risk for the high versus low intake (RR [95% CI] = 1.04 [0.97–1.11]) for cohort studies. A random-effects model was applied to case–control studies and it revealed significantly positive association (OR [95% CI] = 1.18 [1.03–1.34]).

### Subgroup Analyses

#### Menopause Status

In addition, following subgroup analyses, menopause status affected the risk of BC among case–control studies. SFA intake increased the risk among postmenopausal women and was not related to premenopausal women (details shown in Table 2). However, null associations were observed among cohort studies when stratified by menopause status.

**TABLE 2:**
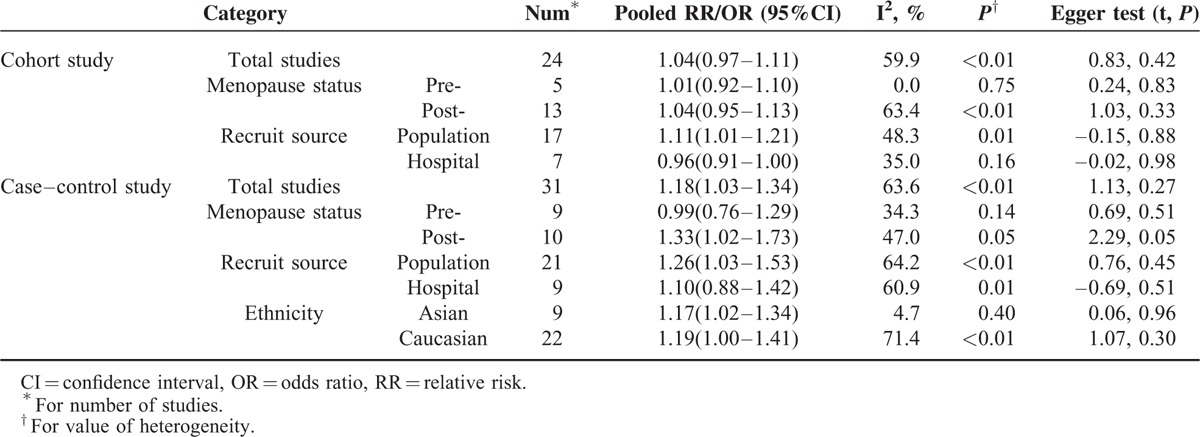
Summary ORs/RRs of Relationship Between Saturated Fatty Acid Intake and Breast Cancer Incidence

#### Recruit Source and Ethnicity

Significant relationship of were observed for the population-based studies (cohort study: RR [95%CI] = 1.11 [1.01–1.21]; case–control study: OR [95%CI] = 1.26 [1.03–1.53]). However, as for hospital-based study, higher SFA intake was not associated with BC risk.

Additionally, only case–control study was conducted by ethnicity. As for cohort studies, most of which were from the same race. Publications suggested that higher SFA intake could increase the risk of BC (Asian: OR [95%CI] = 1.17 [1.02–1.34]; Caucasian: OR [95%CI] = 1.19 [1.00–1.41]).

### Sensitivity Analyses and Publication Bias

Sensitivity analyses were conducted to evaluate the effect of excluding any individual study. By exclusion of 1 literature at a time in turns, summary results of remained literatures did not substantially change.

**FIGURE 4:**
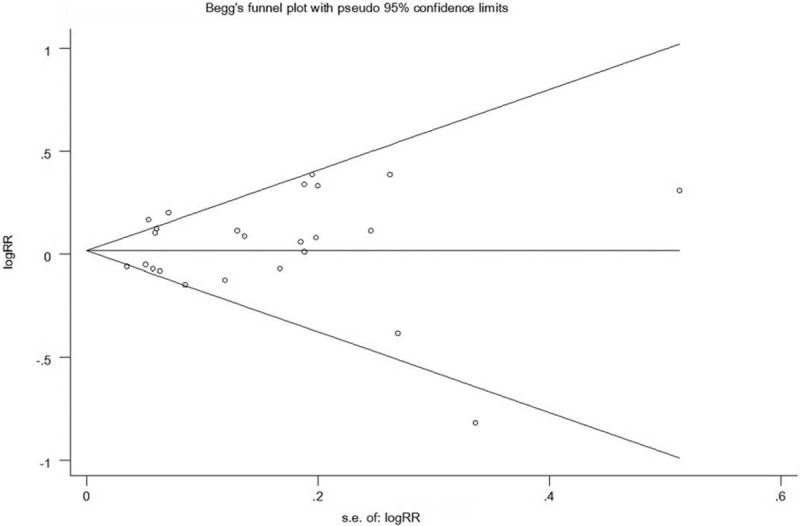
Begg funnel plot for publication bias analyze for cohort study.

**FIGURE 5:**
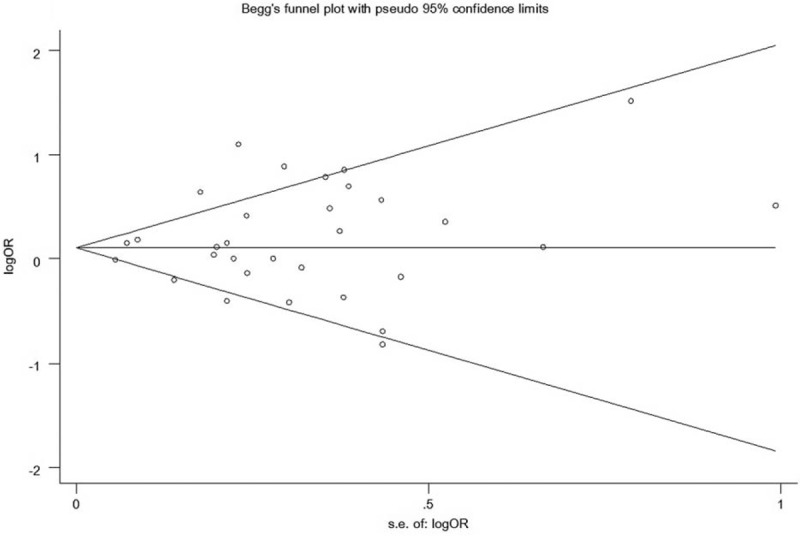
Begg funnel plot for publication bias analyze for case–control study.

Begg funnel-plot and Egger test were used to examine the potential publication bias. All funnel plots indicated no evidence of possible publication bias (shown in Figures 4 and 5). Egger test also showed the lack of publication bias for all studies (shown in Table 2).

## DISCUSSION

In this meta-analysis of observational studies concerning dietary SFA intake and incidence of BC, comparison of high versus low intake of dietary SFA among case–control studies showed that it increased the BC risk while it turned out to be irrelevant among cohort studies. In the following subgroup analyses among case–control studies, we observed positive association in population-based studies along with postmenopausal females. Moreover, when ethnicity was taken into consideration, case–control studies indicated that higher intake of SFA increased the risk of BC.

Compared to the previous publications of meta-analysis, it has both similarities and differences. A previous meta-analysis of prospective studies^8^ arrived the parallel conclusion with the present meta-analysis. Another meta analysis^7^ demonstrated when higher SFA consumption promoted the initiation of BC in both cohort and case-control studies. There were potential reasons for the results. First, in the present study, larger sample size was included and meanwhile literatures were updated. Second, studies used different standards to extract data, so the outcomes differed. Additionally, subgroup-analysis was performed to evaluate menopause status, ethnicity, and study type compared to meta-analysis above.

Micha and Mozaffarian^82^ reviewed RCTs and clarified that specific SFA chain-length had different effects on TC/high-density lipoprotein-cholesterol ratio. Compared to carbohydrate, myristic, palmitic, and stearic acid were not associated with the change of the ratio, but it seemed that stearic acid played a positive role in increasing the ratio. In the EPIC study, Forouhi et al^83^ emphasized the importance of different individual plasma phospholipid SFAs, and found that even-chain SFA (myristic acid, palmitic acid, and stearic acid) increased the risk of disease and odd-chain decreased the risk. In the vitro study, Hardy et al^84^ observed SFA palmitate inhibited BC cells and resulted in the apoptosis, and unsaturated fatty acid promoted the process of proliferation simultaneously. Together, BC is not only related to dietary SFA intake but more associated with the free fatty acid in organism. Determination of internal metabolites of SFA may help us understand the occurrence and development of BC clearly.

Our meta-analysis consists of some limitations. First, only the literatures published in English were included. Several unpublished null articles may be missing because of study with positive results which were searched easier.^85^ Second, possible bias may contain in case–control studies, such as selection bias and recall bias with the contribution of different results of population or hospital-based design. Although, all of our studies considered the confounding factors, such as sex, age, education, BMI, energy intake, smoking, drinking, and nutrient intake, and they reduced the effect of confounding factors to some degree. We still cannot explain potential effects of other dietary habits or behavior and asserted etiology relationship between dietary SFA intake and BC events. The exact mechanisms are not well-established whereby higher SFA intake increasing risk of BC. Since reform and openness, the Chinese have obtained better life conditions and meanwhile their dietary patterns have been changing all the time with tendency to western countries. Hence, we increased our dietary fat intake rapidly, especially saturated fat. Additionally, only 6 articles of subgroup analyses conducted in Asia were referred in our meta-analysis. Small sample size may contribute to the heterogeneity. As for recruit source, due to selection bias, subjects from hospital may result in significant association and different results between case–control and cohort studies. Besides, subjects from hospital are not more representative when compared to the population-based ones.

In conclusion, relationship was found between SFA intake and incidence of BC in case–control studies, and a positive association between higher dietary SFA intake and postmenopausal BC risk was observed in case–control but not in cohort studies. In future, dietary fatty acid intake and serum fatty acid level should be combined to analyze the more detailed relationship with BC.
